# The status of Fas and Fas ligand expression can predict recurrence of hepatocellular carcinoma

**DOI:** 10.1054/bjoc.1999.1065

**Published:** 2000-02-21

**Authors:** Y Ito, M Monden, T Takeda, H Eguchi, K Umeshita, H Nagano, S Nakamori, K Dono, M Sakon, M Nakamura, M Tsujimoto, M Nakahara, K Nakao, Y Yokosaki, N Matsuura

**Affiliations:** Department of Surgery II, Osaka University Medical School, Osaka, 565-0871, Japan; Department of Pathology, School of Allied Health Science, Faculty of Medicine, Osaka University, Osaka, 565-0871, Japan; Diagnostics Research & Development Center, Nichirei Corporation, Tokyo, 189, Japan; Departments of Pathology and Surgery, Osaka Police Hospital, Osaka, 543, Japan

**Keywords:** Fas, Fas ligand, immunohistochemistry, hepatocellular carcinoma, prognostic factor

## Abstract

The status of Fas and Fas ligand (Fas L) expression was investigated in this study for 103 hepatocellular carcinomas (HCC). We studied the expression of the following three factors, Fas and Fas L expression in carcinoma cells and Fas L expression in stromal mononuclear cells (defined as stromal Fas L index). Fas expression in HCC cells was significantly decreased in cases with poor differentiation (*P*< 0.0001) and of larger size (*P* = 0.0058). Fas L expression in carcinoma cells was observed exclusively in moderately or poorly differentiated cases. Furthermore, each factor had prognostic significance for disease-free survival (DFS) (*P*< 0.0001, *P* = 0.0222 and 0.0027 respectively). We then scored the results of each factor and defined the total score as ‘Fas-Fas L risk score’. The *P* -value of the score for DFS was even lower than that of the clinical stage by multivariate analysis. These results suggest that the evaluation of Fas and Fas ligand expression potentially has a significant prognostic value for DFS of HCC patients, in addition to the clinical stage, and can be regarded as a new prognostic marker. © 2000 Cancer Research Campaign


*British Journal of Cancer* (2000) **82**(6), 1211–1217
© 2000 Cancer Research Campaign
Article no. bjoc.1999.1065, available online at http://www.idealibrary.com on IDEAL®

### The status of Fas and Fas ligand expression can predict recurrence of hepatocellular carcinoma

**Y Ito[1,2], M Monden[1], T Takeda[1], H Eguchi[1], K Umeshita[1], H Nagano[1], S Nakamori[1], K Dono[1], M Sakon[1], M Nakamura[3], M Tsujimoto[4], M Nakahara[5], K Nakao[5], Y Yokosaki[2] and N Matsuura[1,2]**

[1]Department of Surgery II, Osaka University Medical School, Osaka 565-0871, Japan; [2]Department of Pathology, School of Allied Health Science, Faculty of Medicine, Osaka University, Osaka 565-0871, Japan; [3]Diagnostics Research & Development Center, Nichirei Corporation, Tokyo 189, Japan; Departments of [4]Pathology and [5]Surgery, Osaka Police Hospital, Osaka 543, Japan

**Summary** The status of Fas and Fas ligand (Fas L) expression was investigated in this study for 103 hepatocellular carcinomas (HCC). We studied the expression of the following three factors, Fas and Fas L expression in carcinoma cells and Fas L expression in stromal mononuclear cells (defined as stromal Fas L index). Fas expression in HCC cells was significantly decreased in cases with poor differentiation ($P < 0.0001$) and of larger size ($P = 0.0058$). Fas L expression in carcinoma cells was observed exclusively in moderately or poorly differentiated cases. Furthermore, each factor had prognostic significance for disease-free survival (DFS) ($P < 0.0001$, $P = 0.0222$ and 0.0027 respectively). We then scored the results of each factor and defined the total score as 'Fas-Fas L risk score'. The *P*-value of the score for DFS was even lower than that of the clinical stage by multivariate analysis. These results suggest that the evaluation of Fas and Fas ligand expression potentially has a significant prognostic value for DFS of HCC patients, in addition to the clinical stage, and can be regarded as a new prognostic marker. © 2000 Cancer Research Campaign

**Keywords**: Fas; Fas ligand; immunohistochemistry; hepatocellular carcinoma; prognostic factor

It has become clear that apoptosis (Kerr et al, 1971), programmed cell death, is related to various diseases (Matsuno et al, 1994; Dowling et al, 1996; Giordano et al, 1997; Strater et al, 1997). It occurs even in carcinoma cells as a common process of cell death, although immortality is one of the important characters of carcinoma cells (Barry et al, 1990; Ling et al, 1993; Tauchi and Sawada, 1994). This has led to intensive study of apoptosis in carcinoma by various approaches. For the mechanism mediating apoptosis, the Fas-Fas ligand (Fas L) (Itoh et al, 1991: Suda et al, 1993) pathway is a prominent candidate. Fas belongs to the tumour necrosis factor receptor family (MW45000) (Itoh et al, 1991: Oehm et al, 1992) and is expressed in various carcinoma cells and cell lines as well as in many normal human cells (Falk et al, 1992; Leithauser et al, 1993: Yoshino et al, 1994). Fas L belongs to the tumour necrosis factor family (MW40000) (Suda et al, 1993; Suda and Nugata, 1994) and is normally expressed by T lymphocytes when they are activated. The signal for cell death is thus transmitted when Fas L binds to Fas on the target cell (Rouvier et al, 1993; Kagi et al, 1994).

The liver is one of the organs which constitutively expresses Fas (Leithauser et al, 1993) and Fas-mediated apoptosis seems to play an important role in inflammation in chronic hepatitis (Hiramatsu et al, 1994; Mochizuki et al, 1996). Furthermore, we previously investigated Fas expression in hepatocellular carcinoma (HCC) and found that, although Fas was expressed in HCC with a high incidence rate, cases with poor differentiation more frequently lacked Fas immunoreactivity (Ito et al, 1998). These findings suggest that the possibility of apoptotic cell death of HCC cells by attack from T lymphocytes via Fas L decreases with the biological aggressiveness of HCC.

Strand et al (1996) investigated Fas L expression in HCC by means of in vitro and in vivo studies. They found that Hep G2 cells, a hepatoma cell line, under stimulus of bleomycin to express Fas L, induced apoptosis of co-cultured Jurkat cells sensitive to Fas-mediated apoptosis, although this event was not observed using Hep G2 cells which do not have the stimulus are negative for Fas L. Furthermore, Jurkat cells plated on cryosections from HCC tissues positive for Fas L mRNA died by apoptosis, whereas they did not turn apoptotic on sections from Fas L mRNA-negative HCC (Strand et al, 1996). These findings indicate that HCC cells, as well as stromal lymphocytes, can express functional Fas L to counterattack and kill the lymphocytes. It is therefore suggested that Fas L, as well as Fas, is an important factor for evaluating the biological aggressiveness of HCC.

All these observations prompted us to investigate the expression of Fas L, as well as Fas, in a large number of HCC to elucidate their clinical significance including their prognostic value. In this study, we investigated the expression of Fas L expression in stromal mononuclear cells and in HCC cells, as well as Fas expression in HCC cells for 103 cases.

*Received 17 March 1999*
*Revised 6 September 1999*
*Accepted 18 October 1999*

*Correspondence to:* N Matsuura, Department of Pathology, Allied Health Science, Osaka University Faculty of Medicine, 1–7, Yamadaoka, Suita, Osaka 565-0871, Japan

### MATERIALS AND METHODS

### Cell lines and tissue specimens

The human colon carcinoma cell line SW480 and pancreatic carcinoma cell line AsPc-1 were provided by the Japanese Cancer

**Table 1** Profile of the 103 HCC cases used in this study

| | | |
|---|---|---|
| Age (years) | | 62.6 ± 10.5 |
| Size (cm) | | 3.8 ± 2.2 |
| Gender | | |
| Male | | 89 |
| Female | | 14 |
| HCV Ab (+/–) | | 70/27 (unknown 6) |
| HBs Ag (+/–) | | 17/86 |
| Liver cirrhosis (+/–) | | 67/36 |
| Portal invasion (+/–) | | 34/69 |
| Intrahepatic metastasis (+/–) | | 23/80 |
| Carcinoma differentiation | | |
| Poor | | 23 |
| Moderate | | 61 |
| Well | | 19 |
| Stage | ≥III | 36 |
| | <III | 67 |

Research Resources Bank. Cells were cultured in RPMI-1640 medium supplemented with 10% fetal calf serum (FCS) at 37°C in 5% carbon dioxide. Tissue specimens were obtained from 103 patients who had undergone surgery for solitary HCC and three patients for breast carcinoma. The tissues were fixed overnight with 10% buffered formalin. After being washed for over 1 h with water and dehydrated through a graded ethanol series at 4°C, the tissues were immersed three times in xylene pools and four times in paraffin pools and embedded in paraffin. The patient profiles are presented in Table 1. Informed consent was obtained from each patient. Four-micrometre-thick sections for each block were prepared for immunohistochemical examination.

### Antibodies

Two kinds of primary antibodies were used in this study. Anti-Fas monoclonal antibody (clone CH-11), the specificity of which had been established previously (Hiramatsu et al, 1994; Mochizuki et al, 1996), was obtained from MBL (Nagoya, Japan). Anti-Fas L polyclonal antibody was from Nichirei (Tokyo, Japan). This antibody specifically recognizes synthetic peptides of the 41st to 54th amino acids located in the intracellular domain of human Fas L. The final dilutions of the primary antibodies were 2.5 $\mu g\ ml^{-1}$ and 10 $\mu g\ ml^{-1}$, respectively.

### Immunohistochemical procedure

Cultured cells were collected, cytocentrifuged onto poly-L-lysine-coated glass slides, immediately fixed in buffered formalin for 1 h, and washed in distilled water for 5 min. Tissue sections were prepared from 4-μm-thick slices from paraffin-embedded specimens. The paraffin was removed in xylene three times, after which the tissues were rehydrated through a graded ethanol series ranging from 100% to 60%.

Endogenous peroxidase activity was blocked with 0.3% hydrogen peroxide ($H_2O_2$) and 0.1% sodium azide in distilled water for 20 min. After three rinsings in phosphate-buffered saline (PBS) pH 7.2, 10% normal rabbit serum (Nichirei, Tokyo, Japan) for anti-Fas antibody or goat serum (Nichirei, Tokyo, Japan) for anti-Fas L antibody was applied for 30 min to block the non-specific reaction. Sections were incubated with anti-Fas antibody or anti-Fas L antibody overnight at 4°C. After rinsing in PBS, they were treated with biotinylated rabbit anti-mouse immunoglobulins (Nichirei, Tokyo, Japan) for anti-Fas or biotinylated goat anti-rabbit IgG (Nichirei, Tokyo, Japan) for anti-Fas L for 30 min. Again after rinsing in PBS, the sections were allowed to react with the avidin–biotin peroxidase complex (Nichirei, Tokyo, Japan). The peroxidase reaction was visualized by incubating the sections with 0.02% 3,3′-diaminobenzidine tetrahydrochloride in 0.05 M Tris buffer (pH 7.6) with 0.01% $H_2O_2$ for 4 min. The sections were counterstained with haematoxylin. Sections for negative control were prepared by using normal mouse serum instead of primary antibody.

The newly established anti-Fas L antibody was subjected to an absorption test to investigate its specificity. The antibody was reacted with a synthetic peptide that this antibody recognizes (final concentration, 50 $\mu g\ ml^{-1}$) at room temperature overnight. Thereafter, the reactant was applied to the sections as primary antibody in an immunohistochemical procedure.

### Immunohistochemical evaluation

We classified the immunohistochemical results into three groups based on Fas expression: ++, more than 80% of HCC cells were clearly positive; +, 10–80% of the cells were positive; –, less than 10% of the cells were positive. To evaluate Fas L expression in the stroma, we observed at least ten fields randomly under a light microscope at × 400 magnification and counted the Fas L-positive cells. The average of the number of Fas L-expressing mononuclear cells per field was defined as the stromal Fas L index. In each case, we carefully checked whether Fas L was expressed in the HCC cells.

Each of these three factors only partially reflects the status of the Fas-Fas L pathway. Therefore, for total evaluation, we scored each factor as follows: (1) Fas (++) or (+) cases were scored as 0 and Fas (–) cases were scored as 1; (2) if the stromal Fas L index was ≥ 2.0, the case was scored as 0, and if < 2.0, it was scored as 1; (3) if Fas L-expressing carcinoma cells were observed, the case was scored as 1, and otherwise, scored as 0. The total amount of the scores for these three factors is named the ‘Fas-Fas L risk score’.

### Survival data

Disease free survival (DFS) data of the 83 patients who underwent curative surgery were analysed. They were followed up from 5 to 73 months (mean 21.0 months). Post-operative DFS curves were constructed by the Kaplan–Meier method.

### Statistical analyses

The values were expressed as mean ± s.e.m. The $\chi^2$ test and Student’s *t*-test were employed for analyses of the immunohistochemical data and clinicopathological parameters such as age, gender, tumour size, liver cirrhosis, TNM stage, degree of differentiation, capsule formation, extracapsular invasion, septal formation, portal invasion and intrahepatic metastasis. Various pathological classifications including degrees of differentiation and stage were based on the classification of the Liver Cancer Group of Japan (1992). Portal invasion and intrahepatic metastasis were histologically diagnosed. Univariate survival data were analysed by the log-rank test. For multivariate analyses for DFS data, we used the Cox proportional hazard model. All *P*-values less than 0.05 were considered to be statistically significant.

 *© 2000 Cancer Research Campaign*

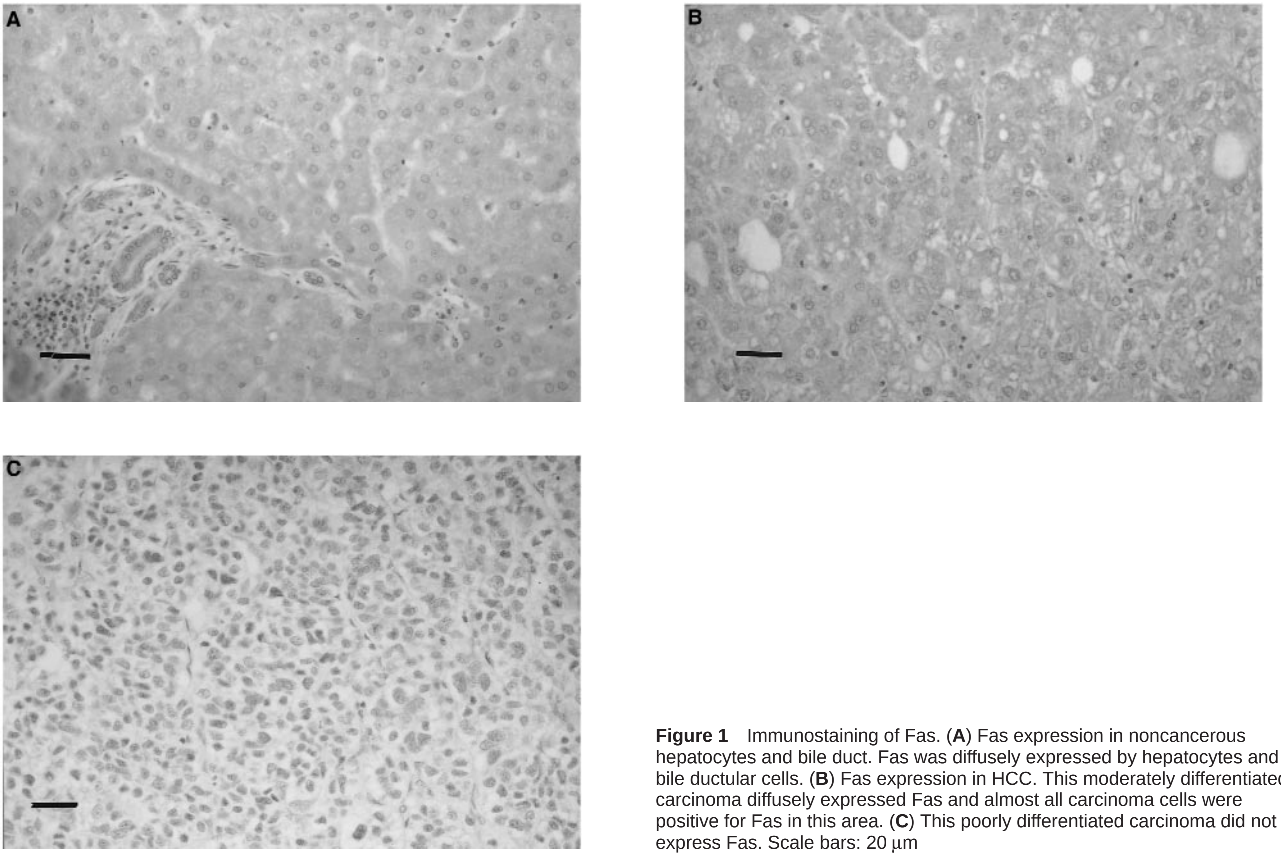

**Figure 1** Immunostaining of Fas. (**A**) Fas expression in noncancerous hepatocytes and bile duct. Fas was diffusely expressed by hepatocytes and bile ductular cells. (**B**) Fas expression in HCC. This moderately differentiated carcinoma diffusely expressed Fas and almost all carcinoma cells were positive for Fas in this area. (**C**) This poorly differentiated carcinoma did not express Fas. Scale bars: 20 μm

**Table 2** Relationship between the factors investigated in this study and clinicopathological parameters

| | 1. Fas expression | | | | |
|---|---|---|---|---|---|
| | ++ | + | – | Total | *P*-values |
| Tumour size (cm) | 3.4±1.9 | 3.8±4.8 | 5.0±2.9 | | 0.0058 |
| Differentiation | | | | | |
| Well | 15 | 4 | 0 | 19 | |
| Moderate | 40 | 17 | 4 | 61 | < 0.0001 |
| Poor | 1 | 5 | 17 | 23 | |
| | 2. Fas L-expressing carcinoma cells | | | | |
| | + | – | Total | | *P*-value |
| Differentiation | | | | | |
| Well | 0 | 19 | 19 | | |
| Moderate | 16 | 45 | 61 | | 0.0398 |
| Poor | 4 | 19 | 19 | | |
| | 3. Fas-Fas L risk score | | | | |
| | 0 | 1 | ≥ 2 | Total | *P*-value |
| Differentiation | | | | | |
| Well | 5 | 14 | 0 | 19 | |
| Moderate | 23 | 27 | 11 | 61 | < 0.0001 |
| Poor | 3 | 5 | 15 | 23 | |

### RESULTS

Fas expression was diffusely or heterogeneously observed in the cell membranes and the cytoplasms of hepatocytes in non-cancerous lesions which were diagnosed as having chronic active or inactive hepatitis with or without liver cirrhosis as well as bile ducts (Figure 1A) and infiltrating mononuclear cells (data not shown). No correlation could be established between Fas expression and various characteristics such as viral infection, active or inactive hepatitis and with or without liver cirrhosis (data not shown). Fas was also expressed in various degrees in HCC cells (Figure 1B). We investigated the relationship between Fas expression in HCC and various clinicopathological parameters. As a result, the absence of or decreased Fas expression in HCC cells was observed significantly more frequently in cases with poor differentiation (Figure 1C) ($P < 0.0001$) and of larger size ($P = 0.0058$) as shown in Table 2.

For the immunohistochemical observation, we used SW480 cells, a colon carcinoma cell line, and AsPc-1 cells, a pancreatic carcinoma cell line as positive controls of Fas L (Figure 2A, B) (Shiraki et al, 1997; von Bernstorff et al, 1999). As negative controls, we employed human breast and liver tissues (Xerri et al, 1997) and confirmed that gland epithelial and myoepithelial cells of mammary glands and hepatocytes were immunohistochemically negative for Fas L (data not shown).

We then investigated Fas L expression in the 103 HCC cases. Fas L immunoreactivity was observed in various degrees in membranes as well as the cytoplasms of the infiltrating mononuclear cells in the stroma adjacent to the carcinoma nests (Figure 2C, D). Infiltrating mononuclear cells were also observed in the non-cancerous portal areas but were only occasionally positive for Fas L (Figure 2E). The stromal Fas L index of each case ranged from 0 to 22.7 (mean ± s.e.m: 2.8 ± 3.8) and was not related to any clinicopathological parameter. Furthermore, Fas L was expressed also in carcinoma cells in 20 of the 103 cases we examined (Figure 2F,G). Sixteen of them were moderately differentiated and the remaining four were poorly differentiated carcinomas (Table 2). Well differentiated carcinomas in our series were all negative for

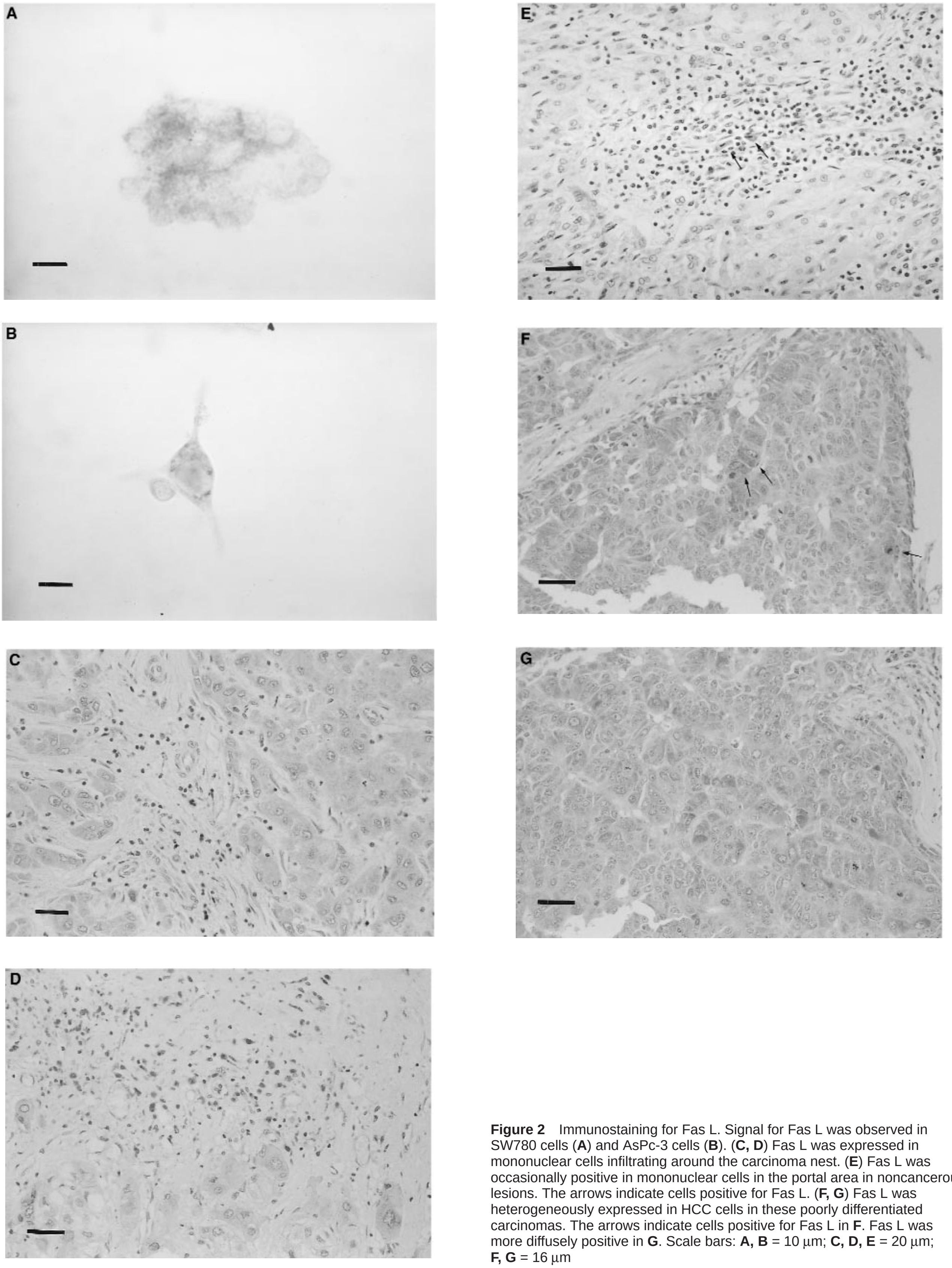

**Figure 2** Immunostaining for Fas L. Signal for Fas L was observed in SW780 cells (**A**) and AsPc-3 cells (**B**). (**C, D**) Fas L was expressed in mononuclear cells infiltrating around the carcinoma nest. (**E**) Fas L was occasionally positive in mononuclear cells in the portal area in noncancerous lesions. The arrows indicate cells positive for Fas L. (**F, G**) Fas L was heterogeneously expressed in HCC cells in these poorly differentiated carcinomas. The arrows indicate cells positive for Fas L in **F**. Fas L was more diffusely positive in **G**. Scale bars: **A, B** = 10 μm; **C, D, E** = 20 μm; **F, G** = 16 μm

 *© 2000 Cancer Research Campaign*

**Table 3** Univariate and multivarate analyses of various parameters for disease-free survival of HCC patients

| | ***P*-values** | | | | | |
|---|---|---|---|---|---|---|
| **Parameters** | **Univariate** | **Multivariate** | | | | |
| Stage (> III vs ≤ III) | < 0.0001 | | | 0.0001 | | 0.0013 |
| Tumour size (≥ 5 cm vs < 5 cm) | 0.0392 | 0.0314 | 0.0768 | | | |
| Differentiation (well, moderate vs poor) | 0.0027 | 0.9141 | 0.2289 | | 0.0690 | |
| Portal thrombus | 0.0181 | 0.6653 | 0.2309 | | | |
| Intrahepatic metastasis | 0.0001 | 0.0520 | 0.2289 | | | |
| Fas expression (++, + vs –) | < 0.0001 | 0.0156 | | 0.0003 | | |
| Stromal Fas L index (≥ 2.0 vs < 2.0) | 0.0027 | 0.0453 | | 0.0241 | | |
| Fas L in carcinoma cells | 0.0222 | 0.0255 | | 0.0396 | | |
| Fas-Fas L risk score (0 vs ≥ 1) | < 0.0001 | | < 0.0001 | | 0.0001 | 0.0001 |

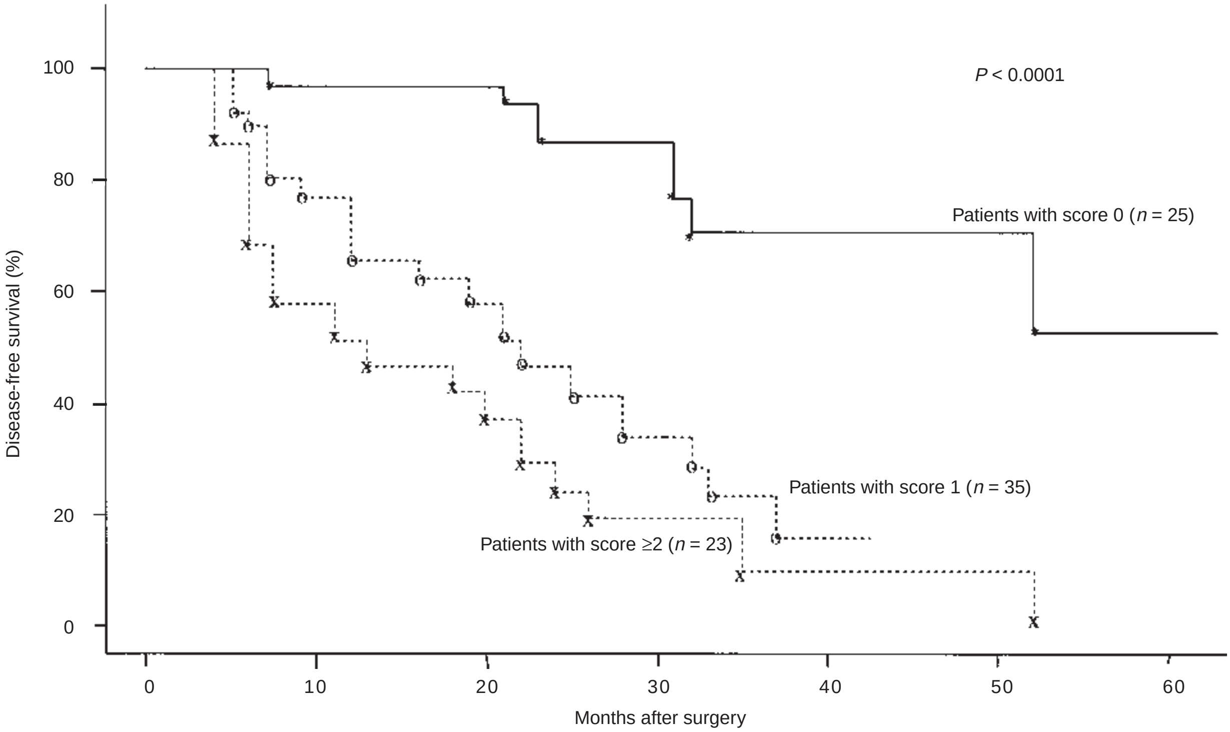

**Figure 3** Disease-free survival curve of 83 patients with Fas-Fas L risk score 0, 1 and ≥ 2 following curative surgery

Fas L in carcinoma cells. No other relationships could be established between the above three factors and clinicopathological features in our series (data not shown).

In the second phase of our study, we investigated the prognostic value for DFS for each of the above three factors by the Kaplan–Meier method. As a result, all three factors, Fas ($P$ < 0.0001), stromal Fas L index ($P$ = 0.0027) and the presence of Fas L-expressing carcinoma cells ($P$ = 0.0222), showed the prognostic impact for DFS by the log-rank test (Table 3).

In order to evaluate the status of Fas and Fas L expression in each case, we defined the Fas-Fas L risk score as mentioned above. The higher the score is, the less likely it would be that the carcinoma cells die with Fas-mediated apoptosis. As shown in Table 1, the score was significantly higher ($P$ < 0.0001) in poorly differentiated carcinomas. Furthermore, patients with a score of 1 or ≥ 2 were much more likely to show recurrence ($P$ < 0.0001) than those with a score of 0 (Figure 3).

We also performed multivariate analysis using the Cox proportional hazard model for DFS (Table 3), together with other parameters which showed the prognostic value for DFS by univariate analyses in our series. As a result, all the three factors, Fas expression in carcinoma cells ($P$ = 0.0156), stromal Fas L index ($P$ = 0.0453) and Fas L expression in carcinoma cells ($P$ = 0.0255) could be regarded as independent prognostic markers for DFS. We then analysed the Fas-Fas L risk score, instead of the three factors by multivariate analyses, with a result that it showed much stronger prognostic impact than the three factors ($P$ < 0.0001 vs 0.0156, 0.0453 and 0.0255). Although the score was strongly

*© 2000 Cancer Research Campaign*

correlated with carcinoma differentiation, it was recognized as a prognostic value independent of carcinoma differentiation, because, when it was analysed with carcinoma differentiation, its *P*-value was 0.0001, whereas that of carcinoma differentiation was over 0.05 (Table 3). Furthermore, when the score was analysed with the clinical stage, its *P*-value was 0.0001, showing that it could be regarded as an independent prognostic factor together with the clinical stage.

### DISCUSSION

We found that non-cancerous hepatocytes, all of which were diagnosed as chronic hepatitis with or without liver cirrhosis, as well as bile ducts very often expressed Fas antigen. Those results are very similar to those of previous studies using the same antibody (Hiramatsu et al, 1994; Mochizuki et al, 1996). Regarding HCC, this and our previous studies (Ito et al, 1998) demonstrated that the absence of or decreased Fas expression in HCC cells was observed significantly more frequently in cases with poor differentiation. Furthermore, cases with lack of Fas expression showed poorer outcomes for DFS. These findings strongly suggest that HCC cells in biologically aggressive cases would less likely die by apoptosis via Fas L, because of their lack of Fas expression in whole or part. Similar results have been reported for lung carcinoma by a previous study with a similar approach (Koomagi and Volm, 1999).

Our immunohistochemical finding that Fas antigen is localized at both the cell surface and cytoplasm agrees with those of studies on Fas-transfected COS cells (Cheng et al, 1994) as well as on the liver using the same antibody (Hiramatsu et al, 1994; Mochizuki et al, 1996). Fas expression in HCC has been investigated using another antibody by other groups but their results did not show mutual agreement (Higaki et al, 1996; Terada and Nakanuma, 1996).

The staining profile of Fas L in each cell should be supported by an absorption test and a recent study demonstrating the presence of Fas L in the cytoplasms of human peripheral monocytes by means of different approaches (Kiener et al, 1997). In our study, Fas L expression in mononuclear cells did not show correlation with any clinicopathological features of HCC, but cases with Fas L expression displayed much better outcomes for DFS. Although further studies are needed to determine whether Fas L expressed by mononuclear cells is always functional, our discovery of the prognostic value of this factor leads to the speculation that Fas L-expressing mononuclear cells act against HCC progression by killing Fas-expressing HCC cells regardless of the characteristics of the carcinoma.

We demonstrated that Fas L was also expressed in HCC cells, in agreement with the findings of other investigators on other carcinomas (Niehans et al 1997; Gratas et al, 1998; Koomagi and Volm, 1999; Loro et al, 1999; Olive et al, 1999; Peduto Eberl et al, 1999; von Benstorff et al, 1999). Regarding the function of Fas L expressed by carcinoma cells, previous in vitro studies using various carcinoma cell lines revealed that it plays a role in counter attacking and killing the activated Fas-sensitive T-cells (O'Connell et al, 1996; Strand et al, 1996; von Bernstorff et al, 1999). Furthermore, Strand et al proved that HCC tissues can express functional Fas L because Jurkat cells sensitive to Fas-mediated apoptosis were killed when they were plated on cryosections from Fas L mRNA expressing HCC (Strand et al, 1996). Our study showed that HCC cases with Fas L-expressing carcinoma cells belonged to moderately or poorly differentiated but not well differentiated carcinomas, and that such cases were significantly more likely to show recurrence. These findings led us to suggest that Fas L expressed by HCC cells is, at least in part, functional and increases the biological aggressiveness of HCC by counter-attacking the stromal lymphocytes.

We defined the Fas-Fas L risk score to totally evaluate the three independent factors: whether HCC cells express Fas, whether the stromal mononuclear cells express Fas L and whether HCC cells express Fas L. Our results indicate that the score accurately reflects the DFS of the patients. In multivariate analysis with carcinoma differentiation, the *P*-value of this score was 0.0001, in spite of being significantly related to carcinoma differentiation, indicating that the prognostic value of this score is not dependent on the characteristics of the carcinoma differentiation. Furthermore, when we performed the analysis at the clinical stage, the *P*-value was also 0.0001 and even lower than that of clinical stage, leading us to conclude that the evaluation of Fas and Fas L expression in HCC tissue can be a novel predictor of recurrence of HCC patients in addition to the clinical stage.

Another interesting and important subject is whether apoptosis in HCC is dominantly induced by the Fas-Fas L pathway. If it is, then we should be able to evaluate the above-mentioned characteristics simply by investigating the apoptotic cell rate in each case. However, a previous study demonstrated that there is no significant relationship between the apoptotic cell rate and the status of Fas and Fas L expression in HCC (Kubo et al, 1998) and we also obtained a similar result (data not shown). Far from that, it has been reported that apoptotic cell rate should reflect the biological aggressiveness of carcinoma including poor prognosis in HCC (Ito et al, 1999) as well as the carcinoma of several organs (Lipponen and Aultomaa, 1994; Vasaslainen et al, 1994; Heatley, 1995; Tormanen et al, 1995; Todd et al, 1996; Yamasaki et al, 1997; Komaki et al, 1998). We therefore hypothesized that there are at least two types of apoptosis in carcinoma. One is an epiphenomenon of the rapid progression of carcinoma, possibly due to the rapid turnover of carcinoma cells. Another is derived from the signal of cell death mainly from the Fas-Fas L pathway. Our results suggest that Fas-mediated apoptosis is not dominant in HCC, although it can be an important phenomenon for evaluating the biological characteristics and prognosis of this carcinoma.

In summary, we have shown that the status of Fas and Fas L expression definitely reflects HCC progression and can serve an independent prognostic factor for DFS of HCC patients. This phenomenon may be useful for future clinical anticancer therapy. For example, stimulating lymphocytes to express Fas L and/or forcing carcinoma cells to express Fas could inhibit HCC progression and reduce the possibility of recurrence.

### REFERENCES

Barry MA, Behnke CA and Eastman A (1990) Activation of programmed cell death (apoptosis) by cisplatin, other anticancer drugs, toxins and hyperthermia. *Biochem Pharmacol* **40**: 2353–2362

Cheng J, Zhou T, Liu C, Shapiro JP, Braner MJ, Kiefer MC and Bar PJ (1994) Protection from Fas-mediated apoptosis by a soluble form of the Fas molecule. *Science* **263**: 1759–1762

Dowling P, Shang G, Raval S, Menonna J, Cook S and Husar W (1996) Involvement of the CD95 (APO-1/Fas) receptor/ligand system in multiple sclerosis brain. *J Exp Med* **184**: 1513–1518

*© 2000 Cancer Research Campaign*

Falk MH, Traugh BC, Debatin K-M, Klas C, Gregory CD, Rickinson AB, Calendar A, Lenoir GM, Ellwart JW and Krammer PH (1992) Expression of the APO-1 antigen in Burkitt lymphoma cell lines correlates with a shift towards a lymphoblastoid phenotype. *Blood* **79**: 3300–3306

Giordano C, Stassi G, Maria RD, Todaro M, Richiusa P, Papoff G, Ruberti G, Bagnasoco M, Testi R and Galluzzo A (1997) A potential involvement of Fas and its ligand in the pathogenesis of Hashimoto’s thyroiditis. *Science* **275**: 960–963

Gratas C, Thoma Y, Barnas C, Taniere P, Hainaut P and Ohgaki H (1998) Up-regulation of Fas (APO-1/CD95) ligand and down-regulation of Fas expression in human esophageal cancer. *Cancer Res* **58**: 2057–2062

Heatley MK (1995) Association between the apoptotic index and established prognostic parameters in endometrial adenocarcinoma. *Histopathology* **27**: 469–472

Higaki K, Yano H and Kojiro M (1996) Fas antigen expression and its relationship with apoptosis in human hepatocellular carcinoma and noncancerous tissues. *Am J Pathol* **149**: 429–437

Hiramatsu N, Hayashi N, Katayama K, Mochizuki K, Kawanishi Y, Kasahara A, Fusamoto H and Kamada T (1994) Immunohistochemical detection of Fas antigen in liver tissue of patients with chronic hepatitis C. *Hepatology* **19**: 1354–1359

Itoh N, Yonehara S, Ishii A, Yonehara M, Mizushima S, Sameshima M, Hase A, Seto Y and Nagata S (1991) The polypeptide encoded by the cDNA for human cell surface antigen Fas can mediate apoptosis. *Cell* **66**: 233–243

Ito Y, Takeda T, Umeshita K, Sakon M, Wakasa K, Matsuura N and Monden M (1998) Fas antigen expression in hepatocellular carcinoma tissues. *Oncol Rep* **5**: 41–44

Ito Y, Matsuura N, Sakon M, Takeda T, Umeshita K, Nagano H, Nakamori S, Dono K, Tsujimoto M, Nakahara M, Nakao K and Monden M (1999) Both cell proliferation and apoptosis significantly predict shortened disease-free survival in hepatocellular carcinoma. *Br J Cancer* (in press)

Kagi D, Ledermann B, Burki K, Seiler P, Odermatt B, Olsen KJ, Podack ER, Zinkernagel RM and Henegartner H (1994) Cytotoxicity mediated by T cells and natural killer cells is greatly impaired in perforin-deficient mice. *Nature* **369**: 31–37

Kerr JFR, Wyllie AH, Currie AR (1971) Apoptosis: a basic biological phenomenon with wide-ranging implications in tissue kinetics. *Br J Cancer* **26**: 239–257

Kiener PA, Dovis PM, Rankin BM, Klebanoff SJ, Ledbetter JA, Starling GC and Liles WC (1997) Human monocytic cells contain high levels of intracellular Fas ligand. Rapid release following cellular activation. *J Immunol* **159**: 1594–1598

Komaki R, Miles L, Ro JY, Fujii T, Perkins P, Allen P, Sikes CR, Mountain CF and Ordonez NG (1998) Prognostic biomarker study in pathologically stage N1 non-small-cell lung cancer. *Int J Radiat Oncol Biol Phys* **40**: 787–796

Koomagi R and Volm M (1999) Expression of Fas (CD95/APO-1) and FAs ligand in lung cancer, its prognostic and predictive relevance. *Int J Cancer* **84**: 239–243

Kubo K, Matsuzaki Y, Okazaki M, Kato A, Kobayashi N and Okita K (1998) The Fas system is not significantly involved in apoptosis in human hepatocellular carcinoma. *Liver* **18**: 117–123

Leithauser F, Dhein J, Mechtersheimer G, Koretz K, Bruderlein S, Henne C, Schmidt A, Debatin KM, Krammer PH and Moller P (1993) Constitutive and induced expression of APO-1, a new member of the nerve growth factor/tumor necrosis factor receptor superfamily, in normal and neoplastic cells. *Lab Invest* **69**: 415–429

Ling YH, Priebe W and Perez SZ (1993) Apoptosis induced by anthracycline antibiotics in P 388 parent and multidrug-resistant cells. *Cancer Res* **53**: 1845–1852

Lipponen PK and Aaltomaa S (1994) Apoptosis in bladder cancer as related to standard prognostic factors and prognosis. *J Pathol* **173**: 333–339

Loro LL, Vintermyr OK, Johannessen AC, Liavaag PG and Jonsson R (1999) Suppression of Fas receptor and negative correlation of Fas ligand with differentiation and apoptosis in oral squamous cell carcinoma. *J Oral Pathol Med* **28**: 82–87.

Matsuno T, Nakagawa K, Sasaki H, Ishine N, Inagaki M, Yagi T, Haisa M, Tanaka N, Sakagami K and Orita K (1994) Apoptosis in acute tubular necrosis and acute renal allograft rejection. *Transplant Proc* **26**: 2170–2173

Mochizuki K, Hayashi K, Hiamatsu N, Katayama K, Kawanishi Y, Kasahara A, Fusamoto H and Kamada T (1996) Fas antigen expression in liver tissues of patients with chronic hepatitis B. *J Hepatol* **24**: 1–7

Niehans GA, Brunner T, Frizelle SP, Liston JC, Salerno CT, Knapp DJ, Green DR and Kratzke RA (1997) Human lung carcinomas express Fas ligand. 57: 1007–1012

O’Connell J, O’Sullivan GC, Collins JK and Shanahan F (1996) The Fas counter attack: Fas-mediated T cell killing by colon cancer cells expressing Fas ligand. *J Exp Med* **184**: 1075–1082

Oehm A, Behrmann I, Falk W, Pawlita M, Maier G, Klas C, Li-Weber M, Richards S, Dhein J and Trauth BC (1992) Purification and molecular cloning of the APO-1 cell surface antigen, a member of the tumor necrosis factor/nerve growth factor receptor superfamily: sequence identify with the Fas antigen. *J Biol Chem* **267**: 10709–10715

Olive C, Chaung C, Nicol D and Falk MC (1999) Expression of apoptotic regulatory molecules in renal cell carcinoma: elevated expression of Fas ligand. *Immunol Cell Biol* **77**: 11–18

Peduto Eberl L, Guillou L, Saraga E, Schroter M, French LE, Tschopp J and Juillerat-Jeanneret L (1999) Fas and Fas ligand expression in tumor cells and in vascular smooth-muscle cells of colonic and renal carcinomas. *Int J Cancer* **31**: 772–778

Rouvier E, Luciani M-F and Golstein P (1993) Fas involvement in $Ca^{++}$-independent T cell-mediated cytotoxicity. *J Exp Med* **177**: 195–200

Shiraki K, Tsuji N, Shioda T, Isselbacher KJ and Takahashi H (1997) Expression of Fas ligand in liver metastases of human colonic adenocarcinomas. *Proc Natl Acad Sci USA* **94**: 6420–6425

Strand S, Hofmann WJ, Hug H, Muller M, Otto G, Strand D, Mariani SM, Stremmel W, Krammer PH and Galle PR (1996) Lymphocyte apoptosis induced by CD95 (APO-1/Fas) ligand-expressing tumor cells: a mechanism of immune evasion? *Nat Med* **2**: 1361–1366

Strater J, Wellisch I, Riedl S, Walczak H, Koretz K, Tandara A, Krammer PH and Moller P (1997) CD95(APO-1/Fas)-mediated apoptosis in colon epithelial cells: a possible role in ulcerative colitis. *Gastroenterology* **113**: 160–167

Suda T and Nagata S (1994) Purification and characterization of the Fas ligand that induces apoptosis. *J Exp Med* **179**: 873–877

Suda T, Takahashi T, Golstein P and Nagata S (1993) Molecular cloning and expression of the Fas ligand, a novel member of the tumor necrosis factor family. *Cell* **75**: 1169–1178

Tauchi H and Sawada S (1994) Analysis of mitotic cell death caused by radiation in mouse leukemia L5178Y cells: apoptosis is the ultimate form of cell death following mitotic failure. *Int J Radiat Biol* **4**: 449–455

Terada T and Nakanuma Y (1996) Expression of apoptosis, proliferating cell nuclear antigen and apoptosis-related antigens (bcl-2, c-myc, Fas, Lewis Y, and p53) in human cholangiocarcinomas and hepatocellular carcinomas. *Pathol Int* **46**: 764–770

Todd D, Yang G, Brown RW, Cao J, D’Agati V, Thompson TS and Truong LD (1996) Apoptosis in renal cell carcinoma: detection by in situ end-labeling of fragmented DNA and correlation with other prognostic factors. *Hum Pathol* **27**: 1012–1017

Tormanen U, Eerola AK, Rainio PO, Vahakangas K, Soini Y, Sormunen R, Bloigu R, Lehto VP and Paakko P (1995) Enhanced apoptosis predicts shortened survival in non-small cell lung carcinoma. *Cancer Res* **55**: 5595–5602

Vasaslainen S, Lipponen P, Talija M and Syrjanen K (1994) Histological grade, perineural infiltration, tumour-infiltrating lymphocytes and apoptosis as determinants of long-term prognosis in prostatic adenocarcinoma. *Eur J Cancer* **30**: 1797–1803

von Bernstorff W, Spanjaard RA, Chan AK, Lockhart DC, Sadanaga N, Wood I, Peiper M, Goedegebuure PS and Eberelein TJ (1999) Pancreatic cancer cells can evade immune surveillance via nonfunctional FAs (APO-1/CD95) receptors and aberrant expression of vfunctional Fas ligand. *Surgery* **125**: 73–84

Xerri L, Devilard E, Hassoun J, Mawas C and Birg F (1997) Fas ligand is not only expressed in immune privileged human organs but is also coexpressed with Fas in various epithelial tissues. *Mol Pathol* **50**: 87–91

Yamasaki F, Tokunaga O and Sugimori H (1997) Apoptotic index in ovarian carcinoma: correlation with clinicopathologic factors and prognosis. *Gynecol Oncol* **66**: 394–400

Yoshino T, Kondo E, Cao L, Takahashi K, Hayashi K, Nomura S and Akagi T (1994) Inverse expression of bcl-2 protein and Fas antigen in lymphoblasts in peripheral lymph nodes and activated peripheral blood T and B lymphocytes. *Blood* **83**: 1856–1861

© 2000 Cancer Research Campaign 
